# Inequalities in Meningioma Survival: Results from the National Cancer Database

**DOI:** 10.7759/cureus.7304

**Published:** 2020-03-18

**Authors:** Andrew I Yang, Kobina G Mensah-Brown, Cole Rinehart, Ramie Fathy, Frederick L Hitti, Jason Brant, John Y.K. Lee

**Affiliations:** 1 Neurosurgery, University of Pennsylvania, Philadelphia, USA; 2 Neurological Surgery, Perelman School of Medicine, University of Pennsylvania, Philadelphia, USA; 3 Otorhinolaryngology - Head and Neck Surgery, University of Pennsylvania, Philadelphia, USA; 4 Otorhinolaryngology, University of Pennsylvania, Philadelphia, USA

**Keywords:** meningioma, survival, prognosis, demographic, socioeconomic

## Abstract

Background

Meningiomas are the second most common primary tumors of the central nervous system. However, there is a paucity of literature examining how healthcare, demographic, and socioeconomic factors impact patient outcomes.

Methods

We conducted a retrospective study of 65,812 patients from the National Cancer Database (NCDB; 2004-2012) who received treatment for their meningioma. Univariate and multivariate analyses were performed with the overall five-year survival as the primary outcome, and the following factors: facility type, geography, housing area, patient insurance, sex, ethnicity, race, income, and education. The multivariate model was adjusted for patient age, co-morbidity, tumor size, behavior, and treatment strategy.

Results

Diagnosis and treatment at an academic/research program, private insurance, female sex, Hispanic ethnicity, and high school diploma conferred a survival advantage on both univariate and multivariate analyses.

Conclusions

Disparities in survival outcomes in patients with meningiomas exist across multiple healthcare, demographic, and socioeconomic factors. Additional research is needed to elucidate the genetic and environmental factors driving these inequalities.

## Introduction

Meningiomas account for 30% of all primary central nervous system (CNS) tumors, with an incidence of 20-40 per 100,000 person-years [1,2]. There are several known predictors of outcomes in meningiomas, including patient age, tumor size or laterality, and surgical resection [1,3,4]. However, there is a paucity of literature examining how healthcare, demographic, socioeconomic factors may impact survival in meningioma patients. This study aimed to determine the effects of treatment facility type, facility location, type of housing area, insurance status, ethnicity, race, sex, income, and education on survival in patients with meningiomas. Specifically, we hypothesized that in patients who received treatment for their meningioma, these factors independently predict survival.

## Materials and methods

Data source

Data were obtained from the National Cancer Database (NCDB; 2004-2012), which includes data from more than 1,500 commission-accredited cancer programs, which in aggregate manage approximately 70% of newly diagnosed cancer cases in the United States [5]. The NCDB is a joint project of the Commission on Cancer of the American College of Surgeons and the American Cancer Society and utilizes the International Classification of Diseases for Oncology (ICD-O) definitions for topography (primary site) and morphology (histology) of neoplasms. The authors note that the NCDB and the hospitals reporting data to the NCDB, which are the source of the data used in the paper, are not responsible for the statistical validity of the data analysis or the conclusions drawn in this study. This study was determined to be exempt by the Institutional Review Board at our institution.

Study population

The NCDB was queried for location codes and histology codes corresponding to meningiomas (9530-9534, 9537-9539). All behavior codes, including benign (0), borderline malignant (1), and malignant (3), were included.

Variables analyzed

Variables investigated were facility type, facility region, facility housing area, insurance status, sex, ethnicity, race, income, and education. Facility type was classified as follows: community cancer program (100-500 newly diagnosed cancer cases per year), comprehensive community cancer program (>500 cases per year), academic/research cancer program (>500 cases per year and at least four postgraduate medical education programs), or integrated network cancer program (owns, operates, leases, or is part of a joint venture with multiple facilities providing integrated cancer care and comprehensive services). Treating facilities were classified by its geographic location in accordance with the US Census Bureau Regions: Northeast, South, Midwest, or West [6]. The housing areas of the treatment facility were defined based on the size of the county in which it resided: metropolitan (>250,000 residents), urban (2,500-250,000 residents), or rural (<2,500 residents). Insurance status was classified into none, Medicare, Medicaid, other government insurance.

Ethnicity was reported as Hispanic or non-Hispanic. Race was reported as white, black, or Asian. Income was determined based on the median household income in the zip code in which each patient reported residence. Finally, education level was determined from the 2012 American Community Survey based on the percentage of adults in the patient's zip code who did not have a high school diploma [7].

Statistical analysis

The overall five-year survival was the primary outcome of interest. Univariate analysis was performed using Kaplan-Meier survival curves and hazard ratios (HRs). The multivariate analysis was performed with the stratified Cox proportional hazards model. The multivariate model included all of the variables described previously. Additionally, it was stratified by the following factors previously known to affect survival: age (0-54, 54-64, 64-74,74-100 years), tumor size (microscopic, <1, 1-2, 2-3, 3-4, 4-5, 5-6, or >6 cm), treatment strategy (surgery, radiation, surgery with adjuvant radiation, and other [surgery with neoadjuvant radiation, surgery with neoadjuvant and adjuvant radiation]), tumor behavior (benign, borderline malignant and malignant), and Charlson-Deyo comorbidity classification (CDCC; 0, 1, 2, 3 or more).

Statistical analyses were performed using Microsoft Open R version 3.3.2 (Microsoft Corp., Redmond, WA). Findings were considered statistically significant if p < 0.05. For HRs, 95% confidence intervals are reported in brackets. Correction for multiple comparisons in the multivariate model was performed using the Benjamini-Hochberg procedure.

## Results

Data were available for 162,222 patients diagnosed with meningioma. After exclusion of patients whose meningiomas were observed without intervention, a total of 65,812 patients (58.8 ± 14.5 years old [mean ± standard deviation]) met the inclusion criteria, with female patients comprising 72% of the cohort. A complete breakdown of demographics is shown in Table 1. The majority of patients had intracranial meningioma, with only 6.4% of the cohort having spinal meningiomas. Nearly all of the cases were benign meningiomas (96.4%). Borderline malignant and malignant meningiomas were reported in only 3.7% of cases. The majority (73.3%) of the cohort underwent surgical resection alone, whereas 17.2% underwent treatment with radiation only.

**Table 1:** Demographic and clinical characteristics of patients with meningioma (NCDB) NCDB, National Cancer Database

Category	Variable	Number (%)
Age	0-54 years	25,171 (38.2)
	54-64 years	16,479 (25.0)
	64-74 years	14,114 (21.4)
	74-100 years	10,040 (15.3)
Facility Type	Academic/Research Program	31,144 (47.3)
	Community Cancer Program	1,573 (2.4)
	Comprehensive Community Care Program	18,866 (28.7)
	Integrated Network Care Program	7,979 (12.1)
Facility Geography	Northeast	12,922 (19.6)
	South	19,645 (29.9)
	Midwest	16,854 (25.6)
	West	10,141 (15.4)
Facility Housing Area	Metro	53,680 (81.6)
	Rural	1,142 (1.7)
	Urban	8,907 (13.5)
Insurance	Private	33,685 (51.2)
	None	2,599 (3.9)
	Medicaid	4,373 (6.6)
	Medicare	23,029 (35.0)
	Other Government	914 (1.4)
	Unknown	1,212 (1.8)
Sex	Female	47,362 (72.0)
	Male	18,450 (28.0)
Ethnicity	Non-Hispanic	57,375 (87.2)
	Hispanic	4,170 (6.3)
	Unknown	4,267 (6.5)
Race	White	53,023 (80.6)
	Black	8,320 (12.6)
	Asian	2,318 (3.5)
	Other/Unknown	2,151 (3.3)
Median Household Income		11,048 (16.8)
	$38,000-47,999	14,594 (22.2)
	$48,000-62,999	17,650 (26.8)
	>$63,000	21,910 (33.3)
Percentage with High School Diploma	>21%	11,397 (17.3)
	13-20.9%	16,569 (25.2)
	7-12.9%	21,098 (32.1)
	<7%	16,171 (24.6)
Tumor Behavior	Benign	63,433 (96.4)
	Borderline Malignant	431 (0.7)
	Malignant	1,948 (3.0)
Tumor Size	Microscopic Focus	24 (0.0)
	<1 cm	1,513 (2.3)
	1-2 cm	8,556 (13.0)
	2-3 cm	11,366 (17.3)
	3-4 cm	9,808 (14.9)
	4-5 cm	7,505 (11.4)
	5-6 cm	5,115 (7.8)
	>6 cm	5,504 (8.4)
Location	Brain	44,984 (68.4)
	Spine	4,195 (6.4)
	Unspecified	13,764 (20.9)
Charlson-Deyo Comorbidity Classification	0	50,604 (76.9)
	1	10,752 (16.3)
	2	3,075 (4.7)
	3+	1,381 (2.1)
Treatment	Surgery Only	48,271 (73.3)
	Surgery + Adjuvant Radiation	3,665 (5.6)
	Radiation Only	11,314 (17.2)
	Other	2,490 (3.8)

Healthcare factors

**Figure 1:**
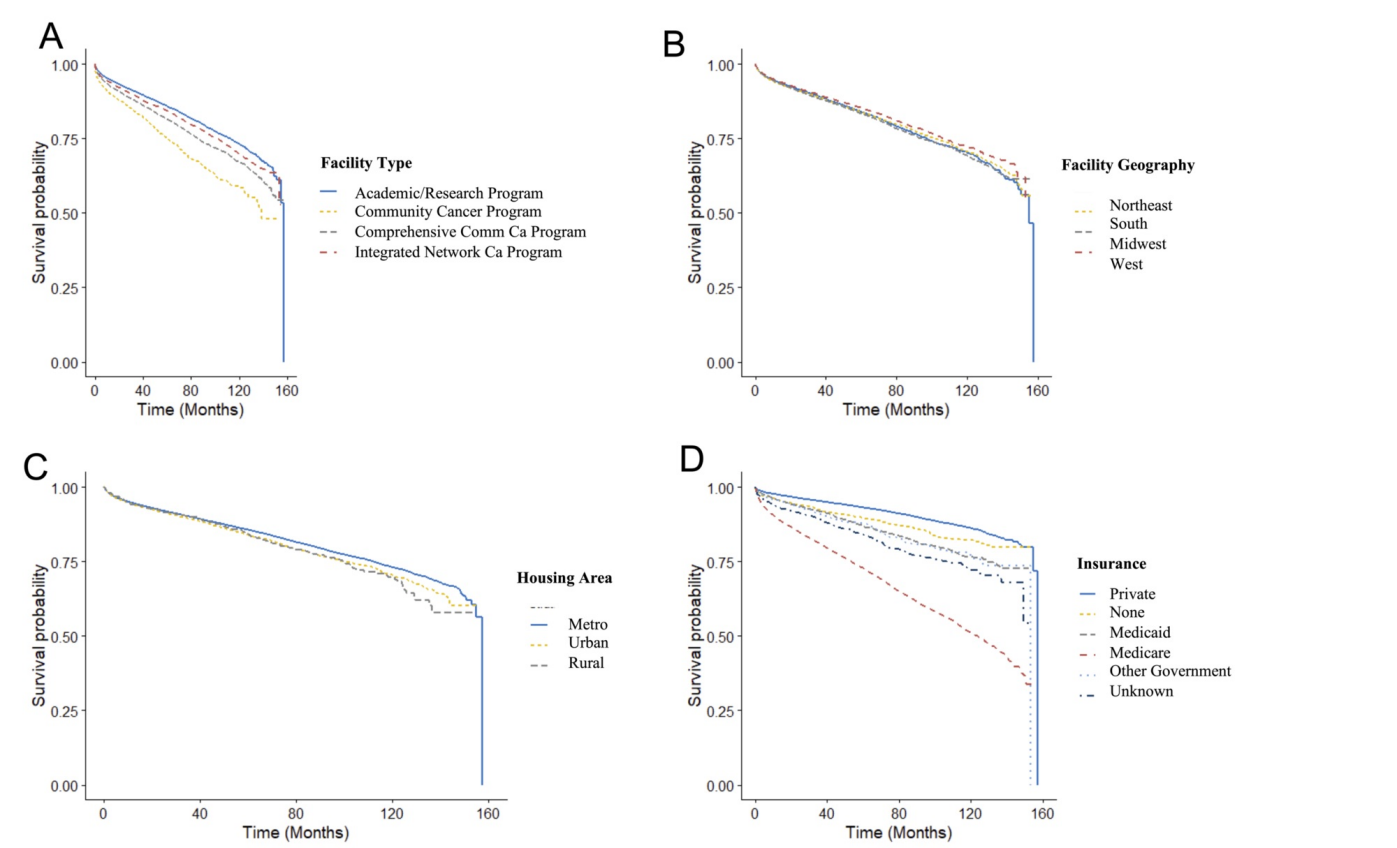
Kaplan-Meier survival curves across facility type (A), facility geography (B), housing area (C), and insurance status (D).

Patients diagnosed and managed at academic/research programs had a statistically significant reduced risk of death in the univariate analysis as compared with patients treated at community cancer programs (HR: 1.82 [1.65-2.00]; p < 0.05), comprehensive community care programs (HR: 1.32 [1.27-1.38]; p < 0.05), and integrated network care programs (HR: 1.14 [1.07-1.20]; p < 0.005) (Figure 1).

Survival differences were found with respect to facility geography. Compared with patients managed at facilities in the northeast, patients managed in the west had a statistically significant reduced risk of death (HR: 0.91 [0.85-0.97]; p < 0.05) in the univariate analysis. In contrast, patients managed at facilities in the south (HR: 0.99 [0.94-1.04]; p = 0.64) or midwest (HR: 1.03 [0.98-1.03]; p = 0.22) had no difference in hazard of death.

Additionally, survival differences were found in terms of housing area of the facility. Compared with patients managed at facilities in metropolitan housing areas, patients managed at facilities in urban housing areas (HR: 1.12 [1.06-1.18]; p < 0.005) had a statistically significant increased risk of death in the univariate analysis. However, rural housing areas (HR: 1.14 [1.00-1.131]; p = 0.06) had no statistically significant difference in the risk of death.

Finally, patients with no insurance (HR: 1.48 [1.30-1.68]; p < 0.005), Medicaid (HR: 1.84 [1.68-2.01]; p < 0.005), Medicare (HR: 4.47 [4.28-4.67]; p < 0.005), and other government insurance (HR: 1.90 [1.59-2.28]; p < 0.005) all had an increased hazard of death compared with patients with private insurance. HR values for each subgroup are shown in Table 2.

**Table 2:** Univariate analysis

Category	Variable	Hazard Ratio	95% Confidence Interval	p-Value
Facility Type	Academic/Research Program	Reference		
	Community Cancer Program	1.82	1.65-2.00	<0.001
	Comprehensive Community Care Program	1.32	1.27-1.38	<0.001
	Integrated Network Care Program	1.14	1.07-1.20	<0.001
Facility Geography	Northeast	Reference		
	South	0.99	0.94-1.04	0.64
	Midwest	1.03	0.98-1.09	0.22
	West	0.91	0.85-0.97	0.003
Housing Area	Metro	Reference		
	Urban	1.12	1.06-1.18	<0.001
	Rural	1.14	1.00-1.31	0.060
Insurance Status	Private	Reference		
	None	1.48	1.30-1.68	<0.001
	Medicaid	1.84	1.68-2.01	<0.001
	Medicare	4.47	4.28-4.67	<0.001
	Other Government	1.90	1.59-2.28	<0.001
Sex	Female	Reference		
	Male	1.68	1.61-1.74	<0.001
Ethnicity	Non-Hispanic	Reference		
	Hispanic	0.63	0.57-0.69	<0.001
Race	White	Reference		
	Black	1.10	1.04-1.17	<0.001
	Asian	0.66	0.58-0.74	<0.001
Income	Reference		
	$38,000-47,999	0.92	0.87-0.97	<0.001
	$48,000-62,999	0.82	0.78-0.87	<0.001
	$63,000+	0.68	0.64-0.72	<0.001
Education	>21%	Reference		
	13-20.9%	1.00	0.95-1.06	0.98
	7-12.9%	0.90	0.85-0.95	<0.001
	<7%	0.75	0.71-0.80	<0.001

Demographic factors

**Figure 2:**
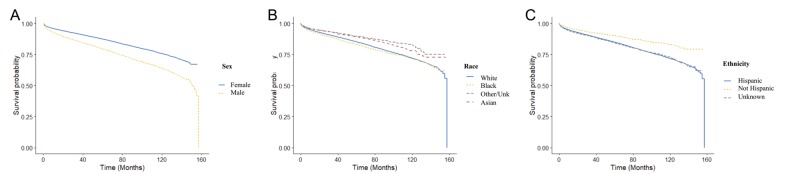
Kaplan-Meier survival curves across gender (A), race (B), and ethnicity (C).

Males had an increased risk of death (HR: 1.68 [1.61-1.74]; p < 0.005) compared with females (Figure 2). Patient race and ethnicity were also associated with a survival difference. Compared with non-Hispanic patients, Hispanic patients had a decreased risk of death (HR: 0.63 [0.57-0.69]; p < 0.005). Similarly, Asian patients (HR: 0.66 [0.58-0.74]; p < 0.005) had a decreased risk of death compared with white patients. In contrast, black patients (HR: 1.10 [1.04-1.17]; p < 0.05) had an increased risk of death compared with white patients in the univariate analysis.

 

Socioeconomic factors

**Figure 3:**
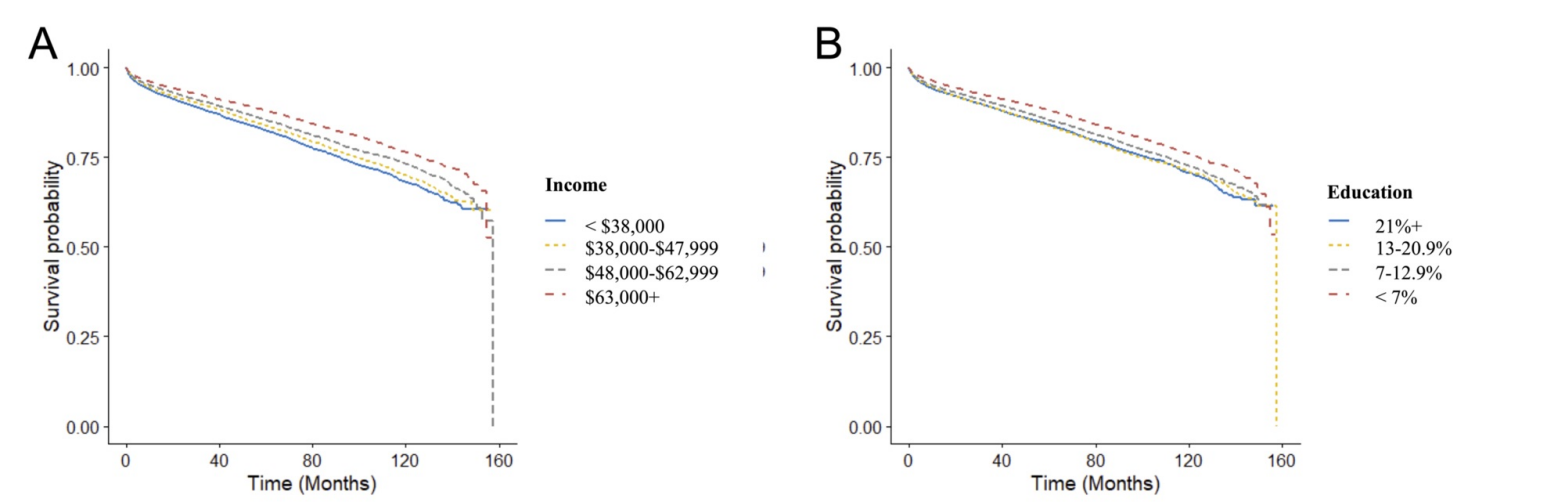
Kaplan-Meier survival curves across income (A) and education (B).

Patients living in zip codes with a median household income of $38,000 to 47,999 (HR: 0.92 [0.87-0.97]; p < 0.005), $48,000 to 62,999 (HR: 0.82 [0.78-0.87]; p < 0.005), and >$63,000 (HR: 0.68 [0.64-0.72]; p < 0.005) all had a decreased hazard of death compared with the lowest income quartile of patients (income < $38,000) in the univariate analysis (Figure 3).

Education differences were also associated with analogous differences in survival. Compared with patients from zip codes where >21% of the population did not have a high school diploma, patients from districts where <7% (HR: 0.75 [0.71-0.80]; p < 0.005) or 7 to 12.9% (HR: 0.90 [0.85-0.95]; p < 0.005) were without a high school diploma had a decreased risk of death. Patients from districts where 13% to 20.9% (HR: 1.00 [0.95-1.06]; p = 0.98) did not have a high school diploma had no statistically significant difference in hazard of death compared with patients in the lowest education quartile.

Multivariate analysis

The multivariate analysis demonstrated findings concordant with the univariate analysis: there was a statistically significant survival advantage for patients managed at an academic/research program, patients with private insurance, female patients, Hispanic patients, Asian patients, patients from regions of higher household income, and those from regions with a higher percentage of high school diplomas (Table 3). Moreover, the multivariate analysis gave congruent results, demonstrating a survival disadvantage in Black patients. In contrast, facilities housing areas and geography were no longer significant predictors of survival.

**Table 3:** Multivariate analysis *Significant after correction for multiple comparisons. **p < 0.05.

Category	Variable	Hazard Ratio	95% Confidence Interval	p-Value
Facility Type	Academic/Research Program	Reference		
	Community Cancer Program	1.31	1.15-1.48	<10^-4^*
	Comprehensive Community Care Program	1.08	1.02-1.14	0.01**
	Integrated Network Care Program	1.08	1.00-1.17	0.05
Facility Geography	Northeast	Reference		
	South	1.00	0.92-1.07	0.8
	Midwest	1.03	0.95-1.11	0.5
	West	1.02	0.94-1.11	0.7
Housing Area	Metro	Reference		
	Urban	0.97	0.90-1.05	0.5
	Rural	0.86	0.72-1.03	0.1
Insurance Status	Private	Reference		
	None	1.42	1.19-1.69	<10^-4^*
	Medicaid	1.70	1.49-1.93	<10^-14^*
	Medicare	1.37	1.26-1.49	<10^-13^*
	Other Government	1.19	0.94-1.52	0.2
Sex	Female	Reference		
	Male	1.44	1.37-1.52	< 10^-15^*
Ethnicity	Non-Hispanic	Reference		
	Hispanic	0.69	0.60-0.79	< 10^-6^*
Race	White	Reference		
	Black	1.10	1.01-1.19	0.02**
	Asian	0.79	0.68-0.93	<10^-2^**
Income		Reference		
	$38,000-47,999	0.93	0.86-1.01	0.08
	$48,000-62,999	0.92	0.84-1.00	0.05
	$63,000+	0.86	0.77-0.95	<10^-2^**
Education	>21%	Reference		
	13-20.9%	1.00	0.92-1.08	0.9
	7-12.9%	0.90	0.83-0.98	0.02**
	<7%	0.80	0.72-0.89	<10^-4^*

After correction for multiple comparisons, patients managed at an academic/research program, patients with private insurance, female patients, Hispanic patients, and patients from regions with a higher percentage of high school diplomas retained a survival advantage. Note that after correction, a survival difference was only noted between extremes for facility type and education: management at academic/research programs conferred a survival advantage only with respect to community cancer programs; patients from districts in the lowest quartile in education had a statistically significant reduction in risk of death only when compared with those from districts in the highest quartile.

Finally, in contrast, median household income and race were no longer significant predictors of survival after correction for multiple comparisons.

## Discussion

This is a retrospective study on the impact of healthcare, demographic, and socioeconomic factors on survival in patients treated for meningiomas based on 65,812 patients treated for meningioma using data available in the NCDB (2004-2012). The majority of patients had benign intracranial meningioma that was surgically resected.

Patients managed at academic/research programs, facilities in the west, and facilities in metro housing areas, patients with private insurance, Hispanic patients, female patients, Asian patients, patients from regions with higher median household income, and those from regions with a greater proportion of residents with a high school diploma had a decreased hazard of death in the univariate analysis. Black patients, in contrast, had an increased hazard of death. Our patient cohort was heterogenous in known predictors of meningioma outcome, patient age, co-morbidity (CDCC), tumor behavior, size, and treatment strategy. We adjusted for these factors with a stratified multivariate model to examine whether healthcare, socioeconomic, and demographic factors were independent predictors of the five-year survival. Although facility geography and housing area were no longer significant in the multivariate analysis, we saw congruent results for the remaining factors.

Although healthcare, demographic, and socioeconomic factors have been reported to affect a variety of outcomes for patients afflicted by cancer, minimal literature exists detailing the effects of these factors on survival in meningioma patients.

Healthcare factors

McCarthy et al. found a survival advantage for patients with benign meningiomas treated at academic medical centers in a cohort of approximately 9,000 patients from the NCDB (1985-1988 or 1990-1992) [4]. Similarly, in an analysis of 14,239 patients with intracranial meningiomas undergoing surgery in a single state, McKee et al. reported lower 30-day mortality at high-volume centers [8]. Our data corroborate these findings in a larger cohort of patients in identifying a survival advantage for those managed at academic/research programs but only in comparison with community cancer programs. This survival benefit may be in part due to a larger percentage of patients who undergo definite initial treatment versus observation in academic/research programs compared with community cancer programs [9].

Insurance status has also been associated with differences in patient outcomes. McKee et al. reported that Medicare and Medicaid insurance were associated with higher 30-day mortality [8]. We report similar findings, with decreased survival in patients with federal insurance compared with those with private insurance. No literature to the authors’ knowledge previously examined facility geography or housing area with regard to survival outcomes in meningioma patients. These were not found to be associated with patient outcomes in our multivariate analysis.

Demographic factors

In this study, female patients were found to have a decreased hazard of death compared with male patients. In an analysis of 12,284 patients from the Surveillance, Epidemiology, and End Results (SEER) database, Cahill and Claus also reported a decreased hazard of death in female patients with non-malignant intracranial meningiomas [3]. Based on data from the Central Brain Tumor Registry of the United States, Achey et al. likewise found a survival benefit in females in non-malignant meningiomas [1]. On the contrary, other studies did not find differences in outcomes based on sex [4,8]. Moreover, Ambekar et al. found that female patients actually had increased odds of adverse events (defined as in-hospital death or discharge to a facility other than home) following surgery, although this study was based on 13,792 patients with spinal meningiomas (Nationwide Inpatient Sample, 2001-2010) [10].

Our data showed that Hispanic patients had a decreased hazard of death compared with non-Hispanics. In the aforementioned studies, there were no difference in outcomes between Hispanics and non-Hispanics [1,4,8] However, favorable outcomes in Hispanic patients have been reported for other primary CNS tumors; for example, Farah et al. demonstrated increased survival rates in Hispanics in a cohort of 33,204 patients with glioblastoma [11]. Similar observations have led to the term “Hispanic paradox,” whereby Hispanic patients experience better outcomes despite lower socioeconomic status and decreased access to healthcare. Although this phenomenon has been validated in a variety of different diseases, the cause is largely unknown and is likely driven by a variety of factors [12].

Our data demonstrated that Asian patients had a decreased hazard of death compared with white patients, whereas black patients had an increased hazard of death in the univariate analysis [13]. Black race has been reported to be a negative prognosticator of outcomes in several of the aforementioned studies [1,3,10]. Notably, Achey et al. included white, black, and Asian/pacific islanders in their study and found that black patients had the worst outcomes [1]. Furthermore, Cahill and Claus reported that black patients were less likely to undergo surgical treatment, which was a positive predictor in their multivariate model [3]. However, in our stratified multivariate model, after accounting for multiple comparisons, race did not demonstrate any association with patient outcomes. This suggests that known predictors of outcomes for which we stratified, such as CDCC and treatment strategy, may account for racial differences in patient outcomes seen in prior studies.

Socioeconomic factors

We found that patients living in a zip code with >21% of residents with high school diplomas have a decreased hazard of death when compared with those in districts where <7% of residents have a high school diploma. Although it has been widely reported that patients with a lower socioeconomic status have worse health outcomes, including for brain tumors in general, the literature pertaining to meningiomas is extremely limited [14]. In a single-center study of 281 patients with intracranial meningioma, Nayeri et al. found that factors associated with low socioeconomic status, including Medicaid coverage and lack of college degree, were predictors of poor patient follow-up after resection [15].

Limitations

Limitations of this study are inherent to a retrospective database analysis. Specifically, as data in the NCDB are compiled from a number of institutions, its accuracy is limited by inconstancies in data collection and recording across participating institutions. Notably, cause of death is not recorded in the NCDB. Finally, income and education level were determined based on the population of the patient’s zip code, which may not necessarily correspond to the individual patient characteristics.

## Conclusions

Treatment at an academic/research program, private insurance, female sex, Hispanic ethnicity, and a high school diploma were associated with a decreased hazard of death in patients with meningiomas that were treated. Additional research is needed to confirm these findings and to elucidate potential genetic and environmental drivers of these disparities in health outcomes.
